# Asymmetric Molecular Adsorption and Regioselective Bond Cleavage on Chiral PdGa Crystals

**DOI:** 10.1002/advs.202309081

**Published:** 2024-02-14

**Authors:** Nestor Merino‐Diez, Raymond Amador, Samuel T. Stolz, Daniele Passerone, Roland Widmer, Oliver Gröning

**Affiliations:** ^1^ Nanotech@surfaces Laboratory Empa – Swiss Federal Laboratories for Materials Science and Technology Überlandstrasse 129 Dübendorf 8600 Switzerland

**Keywords:** asymmetric catalysis, density function theory, intermetallic compound, scanning tunneling microscopy, scanning tunneling spectroscopy, surface chemistry

## Abstract

Homogenous enantioselective catalysis is nowadays the cornerstone in the manufacturing of enantiopure substances, but its technological implementation suffers from well‐known impediments like the lack of endurable catalysts exhibiting long‐term stability. The catalytically active intermetallic compound Palladium‐Gallium (PdGa), conserving innate bulk chirality on its surfaces, represent a promising system to study asymmetric chemical reactions by heterogeneous catalysis, with prospective relevance for industrial processes. Here, this work investigates the adsorption of 10,10′‐dibromo‐9,9′‐bianthracene (DBBA) on the PdGa:A($\bar{1}\bar{1}\bar{1}$) Pd_3_‐terminated surface by means of scanning tunneling microscopy (STM) and spectroscopy (STS). A highly enantioselective adsorption of the molecule evolving into a near 100% enantiomeric excess below room temperature is observed. This exceptionally high enantiomeric excess is attributed to temperature activated conversion of the S to the R chiral conformer. Tip‐induced bond cleavage of the R conformer shows a very high regioselectivity of the DBBA debromination. The experimental results are interpreted by density functional theory atomistic simulations. This work extends the knowledge of chirality transfer onto the enantioselective adsorption of non‐planar molecules and manifests the ensemble effect of PdGa surfaces resulting in robust regioselective debromination.

## Graphical abstract

The smell of D‐limonene (left‐handed) and L‐limonene (right‐handed) is a famous example how chirality impacts physiological function. The former smells of oranges whereas the latter of turpentine, highlighting the importance of chiral selectivity. they have investigated highly chiral selective adsorption and halogen abstraction of 10,10′‐dibromo‐9,9′‐bianthracene molecules on the surface of the chiral intermetallic compound Palladium‐Gallium by Scanning Tunneling Microscopy.

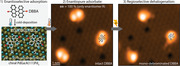

## 1 Introduction

Chirality characterizes our universe over many length scales from the weak interaction among elementary particles, through molecular and crystal enantiomers, to spiral galaxies.^[^ ^1^ ^]^ The crucial relevance of chirality in nature manifests in vital daily aspects, like physiologically active molecules in our body, ruled, for example, by *left‐handed* proteins and *right‐handed* sugars,^[^ ^2^ ^]^ or pharmaceuticals where metabolizing the wrong enantiomeric form of a drug can produce tragic side effects.^[^ ^3^, ^4^ ^]^ For targeting the desired chirality, mastering the production of enantiopure products constitutes a global goal particularly for pharmaceutical, agrochemical, or food purposes. Besides the well‐known role of chirality in biochemistry, the development of chiral solid materials flourishes rapidly nowadays and the current impact of chiral technology applies in non‐linear optics,^[^ ^5^ ^]^ smart coatings,^[^ ^6^ ^]^ or chemical biosensoring^[^ ^7^ ^]^ among other areas.^[^ ^8^ ^]^

Heterogeneous asymmetric catalysis stands out among the most efficient technological approaches to achieve enantioselective industrial manufacturing, particularly by the use of catalytic solid surfaces given its numerous advantages. These benefits include an easy separation of products from the catalyst, a facilitated reuse and regeneration of the latter, a drastic reduction in metal traces contamination, or a wider range of operability in terms of temperature, pressure, and chemical or mechanical stability.^[^ ^9^ ^]^ Up to date, relatively low enantioselective yields in comparison with homogeneous catalysis still prevent heterogeneous asymmetric synthesis from extensive industrial application. Recent advances in solid‐phase chemistry aim to remedy this shortage of chiral transfer from the catalytic solid substrate.^[^ ^10^ ^]^ Main efforts are devoted to the functionalization of surfaces with organic chiral ligands, both of oxides and metallic surfaces.^[^ ^11^ ^]^ Nevertheless, these commonly expensive ligands do often not provide competitive enantioselectivity yields and their delicate chemical stability drastically narrows down their operability conditions. Therefore, the direct use of non‐functionalized surfaces of solids provides greater catalyst robustness. The need of (partially‐)delocalized electrons largely excludes chiral insulators like quartz from the catalogue of possible options, as their large band gap prevents valence states to engage in complex catalytic processes.^[^ ^12^ ^]^ Hence, highly active catalytic substrates ideally should exhibit metallic electrons. Unfortunately, most conventional metallic surfaces are achiral. Although numerous experiments achieved enantioselectivity on chiral “kinked‐steps” of high Miller index surfaces of coinage metallic crystals,^[^ ^13^, ^14^, ^15^, ^16^, ^17^, ^18^ ^]^ the abundance of chiral active centers on these substrates is commonly relatively low compared to the total area of the surface, yet being mostly achiral and so yielding overall low enantioselectivity. The convenience of chiral surfaces with high or complete enantioactive areas is thus self‐demanded.

The chiral intermetallic compound PdGa, preserving intrinsic bulk chirality until top‐layer terminations, represents a direct manifestation of innate chirality on its surfaces along extensive and homogenous macroscopic areas. The intrinsic chirality of cubic PdGa originates from the structure its bulk unit cell belonging to the non‐centrosymmetric space group P2_1_3, and therefore existing in two enantiomeric forms denoted as PdGa:A and PdGa:B. Bulk‐truncated surfaces can be prepared atomically flat and well‐defined with nanoscale precision by standard ultra‐high vacuum methods, without any further modification. The catalytic potential of PdGa is evidenced by recent experiments illustrating the enantioselective adsorption of prochiral molecules,^[^ ^19^, ^20^ ^]^ different on‐surface asymmetric reactions^[^ ^19^, ^21^, ^22^ ^]^ and the transmission of its surface chirality into unidirectional molecular motion.^[^ ^23^ ^]^ Moreover, due to the partially covalent character of PdGa chemical bonding, the simultaneous presence of localized and delocalized electrons can give rise to the formation of exotic topological phenomena.^[^ ^24^ ^]^

PdGa's bulk chirality is transferred along all crystallographic orientations and so preserved at all surfaces, each of them feature a particular geometrical arrangement of the very top‐layer atoms ranging from equally‐separated Pd monomers or trimers, to unidirectional Pd rows.^[^ ^25^ ^]^ The unusual corrugated atomic structure of these top‐layer PdGa terminations results into an ordered separation and confinement of the catalytically active sites along the surface, in contrast with elemental metallic surfaces where a flat compact arrangement of top‐layer atoms results into a homogeneous and continuous site distribution. The striking influence that the modulated catalytic activity of PdGa surfaces can impose on the reactivity of different adsorbates is known as ensemble effect.^[^ ^26^, ^27^ ^]^ Moreover, the relatively open surface structure of PdGa top‐layer terminations allows bulk chirality of deeper layers to crop up and strongly influence on‐surface molecular adsorption and reaction processes. All together, the ample catalogue of PdGa surfaces, combining bulk chirality with a pronounced ensemble effect, presents as an alluring workbench to study disparate on‐surface enantioselective phenomena including catalytic processes.

In this work, we focus on the PdGa:A($\bar{1}\bar{1}\bar{1}$) Pd_3_‐terminated surface as substrate, featuring equally‐separated trimers of palladium atoms as top‐layer termination. By means of scanning tunneling microscopy and corroborated by density functional theory calculations, we investigate the chirality transfer of PdGa onto DBBA, a well‐known molecule for its pioneering role in the on‐surface synthesis of atomically‐precise graphene nanoribbons.^[^ ^28^, ^29^ ^]^

## 2 Results

**Figure 1:**
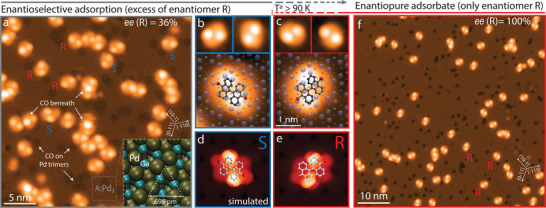
Temperature‐dependent enantioselective adsorption of DBBA on A:Pd_3_. a) Enantioselective phase formation featuring an enantiomeric excess of enantiomer R after cold‐deposition at 90 K. Letters indicate examples of each enantiomer in its three favored orientations. White arrows point to CO molecules beneath some DBBA anthracene units and adsorbed on top‐layer Pd trimers (hollow sites). Corner inset: A:Pd_3_ surface model. b) Enantiomer S and c) R in their threefold symmetric adsorption orientations. and d,e) their corresponding simulated STM appearances. Adsorption models superimposed in (b–e) as visual guide. f) Enantiopure phase featuring exclusively enantiomer R after warming the sample up to 160 K for 25 min, and then cooling to 5 K. [Tunn. parameters: a–c) V = 1.00 V, I_t_ = 20 pA f) V = 50 mV, I_t_ = 15 pA].

DBBA can accommodate into chiral geometries upon adsorption onto the solid surface of a crystal. Molecule and surface form a 3D chiral system in a process known as “adsorption‐induced chirality.”^[^ ^30^ ^]^ **Figure** **1**a summarizes the observed features for a sub‐monolayer DBBA deposition at 90 K (imaged at 5 K) on PdGa:A($\bar{1}\bar{1}\bar{1}$)Pd_3_, referred here on as A:Pd_3_ surface for simplicity. We observe the DBBA in two different adsorption configurations, each in three 120° rotated variants expected from the threefold symmetry of the substrate. We identify the two configurations to be related to the R and S enantiomeric form of the DBBA. In this particular system, chirality manifests by the relative out‐of‐plane orientation of DBBA anthracene subunits with respect to the surface lattice. These subunits are not adsorbed completely planar but partially tilted with respect to the surface, as observed previously in different systems^[^ ^31^, ^32^ ^]^ (see Figure S1, Supporting Information). The STM signature of the DBBA molecules features a characteristic oval shape with two protruding lobes corresponding to the anthracene termini pointing away from the surface (Figure S2, Supporting Information). This appearance together with the orientation of DBBA molecules with respect to PdGa crystallographic orientations, allows for the unambiguous identification of the two chiral conformers R/S as depicted in Figure 1b–e. We assign the (R‐) S‐labeling according to the apparent (counter‐)clockwise helicity of each enantiomeric form when imaged by STM. At 90 K, we determine an enantiomeric excess (*ee*  = |*R* − *S*| *100/(*R* + *S*)) of 36% for the R enantiomer, depicting a partially enantioselective adsorption scenario already at low temperature deposition.

Already upon mild annealing in the range between 100 and 200 K for a few minutes, we observe the evolution of an essentially enantiopure phase (*ee*  =  100%), where only the R conformer persists as displayed in Figure 1f (see Figure S3, Supporting Information). The disappearance of the S enantiomer at higher temperatures raises the question about the whereabouts of this energetically non‐favored conformer. Here, we consider the following three possible explanations. First, we can discard a selective desorption of the S enantiomer because the adsorbate coverage does not change after the annealing. Second, the enantiomeric form S could turn into R through an anti‐parallel rotation around the common C─C bond linking both anthracene subunits. Nevertheless, being DBBA on PdGa an atropisomer^[^ ^33^ ^]^ (stereoisomers emerging from hindered rotations, also referred in literature as axial chirality), this anthracenes' transposition is energetically impeded because the steric hindrance increases exponentially as the dihedral angle between the two anthracenes approaches zero (see Figure S4, Supporting Information). This energetically unaffordable steric hindrance originates essentially from the spatial proximity of the four hydrogen atoms located in‐between the anthracene units which, to complete this anti‐parallel rotation, should neatly overlap their atomic nucleus along the process. For this reason, before R to S conversion, molecular fragmentation is anticipated in this process as reported previously for molecules with the same carbon skeleton.^[^ ^34^ ^]^ Rejecting a selective desorption of the S enantiomer, and an anti‐parallel rotation of its anthracenes, we conclude that the S to R conversion must proceed through a series of conformational changes on the surface avoiding high steric hindrance situations.

**Figure 2:**
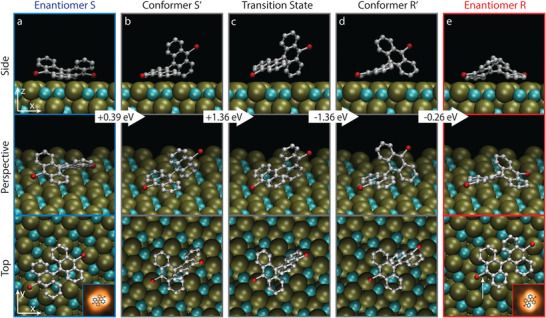
Enantiomer‐flipping model on A:Pd_3_. Minimum‐energy‐path model resulting from NEB‐DFT calculations (48 replicas) illustrating how a) enantiomer S undergoes a b–d) series of surface‐assisted conformational changes via a transition state and the proximity states (S' and R') until turning into e) enantiomer R. Arrows in between columns indicate energy barriers between consecutive stages of the flipping process. Corner insets: STM image of corresponding enantiomers S/R. White arrow in *e* indicates position for regioselective dehalogenation.

As we cannot observe this dynamics directly in experiment, we run minimum‐energy‐path ab‐initio calculations using the nudged elastic band method to elucidate its details. Enantiomer S can transform into R through an energetically favored sequence of surface‐assisted molecular changes illustrated in **Figure** **2**. DBBA molecules synchronize a parallel rotation of both anthracene units with a simultaneous translation over the surface along this step. Starting from the enantiomeric form S (Figure 2a), the adsorbate places one anthracene planar onto two adjacent Pd trimers and lifts the other anthracene from the surface (Figure 2b). This lifting continues until the anthracene unit almost reaches verticality with respect to the surface (Figure 2c), featuring the rate‐determining transition state at this point. This high energy barrier (1.36 eV) remains energetically far from the calculated desorption energy (2.60 V) of DBBA molecules on the A:Pd_3_ surface. Due to intrinsic limitations of the NEB (nuged elastic band) method, which captures a too “direct” transition path discretized into relatively few intermediate configurations, the barrier value obtained by DFT (density functional theory) can be considered significantly too large and presents an upper limit. High level ab initio molecular dynamics calculations targeted to the description of the free energy landscape (metadynamics) would be needed to quantitatively obtain a more realistic value in concordance with experiments. In particular, we hypothesize an intermediate state between the two enantiomeric forms (at slightly higher energy with respect to R and S, i.e., not observable experimentally) and advanced methods would be necessary to estimate the (free) energy barriers from and to this intermediate. After this uplifting, anthracenes tilting changes with respect to the chiral surface lattice and so the molecule already feature an R‐like molecular conformation (Figure 2d). Finally, the lifted anthracene relaxed toward the surface and the other anthracene by contrast, which remained adsorbed planar atop two adjacent Pd trimers along the whole process, detaches partially from the surface (Figure 2e). Finally, the adsorbate adopts precisely the enantiomeric form R. Experimentally, we have checked that this enantiomeric flipping over the surface is attainable not only by thermal excitation (Figure 1) but also by STM tip‐manipulation of individual molecules (see Figure S5, Supporting Information, and Experimental Section for experimental details). Note that the NEB‐DFT calculation shows a slight energetic favor for the S enantiomer, which is at odds with the experiment. Whereas DFT is reliable to find the local adsorption configurations correctly, the ability of accurately describing dispersive interactions in the intermediate distance regime (between 3 and 5 Angstrom) with the required precision of some 50 meV is a difficult issue. This is particularly true in presence of mixed physisorption/chemisorption of large molecules and complex surface structure as is the case here.^[^ ^35^ ^]^

**Figure 3:**
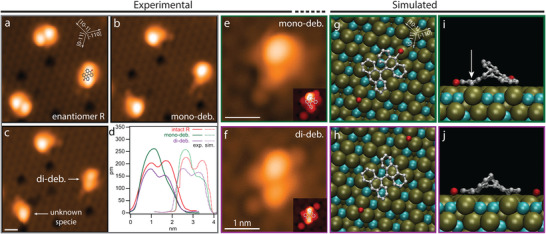
Tip‐induced debrominated DBBA species on A:Pd_3_. a–c) STM images showing a) intact, b) mono‐, and c) di‐debrominated DBBA molecules and d) experimental and simulated height profiles of each specie. e,f) Zoom in e) mono‐ and f) di‐debrominated DBBA molecules, and g–j) optimized‐adsorption models from g,h) top and i,j) side views. Insets in (e,f) are simulated STM images originated from (g,h) models. White arrow in (i) indicates regioselective dehalogenated position. [Tunn. parameters (V,I_t_) : a–c, e, f) 50 mV, 30pA].

To get insight on the impact of the enantioselective adsorption on possible reaction processes, we study the tip‐induced mono‐ and di‐debromination of the DBBA molecules, interestingly discovering the first debromination event to be a nearly perfect regioselective process. Specifically, a voltage induced excitation by the STM tip above a certain threshold always results into the scission of one specific of the two C─Br bonds, which in relation to the substrate are inequivalent. This is the case even when positioning the tip on top of the non‐favored C─Br bond. **Figure** **3a,b** illustrates this bond selectivity, showing R enantiomers before and after tip‐induced mono‐debromination in its three equivalent energetically favored orientations (see Figure S6, Supporting Information). Once DBBA molecules are debrominated, they feature a different STM signature, easily distinguishable from the non‐debrominated molecule. First, mono‐debrominated molecules feature a single protruding lobe, higher than the two of intact DBBA molecules (see height profiles in Figure 3d). This indicates that the non‐debrominated anthracene subunit is uplifted and adopts a more perpendicular tilting with respect to the surface. This conformation reduces strain which stems from the debrominated unit absorbing significantly closer to the surface upon bromine cleavage. This scenario is confirmed by DFT calculations, which reveal that the distance between the radical carbon (see white arrow in Figure 3i) and the closest atom of the A:Pd_3_ substrate – in this instance, a second‐layer Ga species – reduces from 3.71 Å in the intact DBBA molecule, to 2.08 Å after debromination. The closest Pd atom atom to the radical C, by comparison, features distances of 3.32 Å in the intact molecule versus 2.46 Å after the debromination. This reduced distance of the radical carbon with respect to the Ga species of the metallic surface, matches the values expected for organometallic coordination,^[^ ^36^, ^37^, ^38^ ^]^ and indicates a chemisorption scenario as observed previously for dehalogenated DBBA molecules on coinage metals.^[^ ^32^, ^39^, ^40^, ^41^ ^]^ Finally, di‐debrominated molecules restore the two‐lobed appearance similar to intact DBBA yet featuring lower overall height, precisely resembling di‐debrominated DBBA molecules reported previously on Cu(111).^[^ ^32^ ^]^ DFT calculations show that this second debrominated carbon similarly reduces its distance to the closest atom of the A:Pd_3_ surface. The closest substrate atom to the radical C is, in this instance, of the Pd species, and this distance reduces from 3.63 in the intact molecule to 2.18 Å after the debromination. For comparison, the distance to the closest Ga species is 4.02 Å in the intact case, versus 3.11 Å after the debromination. We observe an excellent matching between experimental and simulated STM images for the proposed adsorption registry for all species. As single Br atoms adsorb preferentially on top of the Pd trimers, cleaved Br atoms remain generally next to the debrominated molecules (see Figure S6, Supporting Information). This preferred adsorption of single Br atoms onto Pd trimers limits the average dissociation distance of cleaved Br atoms to ≈ 270 pm and ≈ 430 pm for the first (regioselective) and second debromination, respectively.

**Figure 4:**
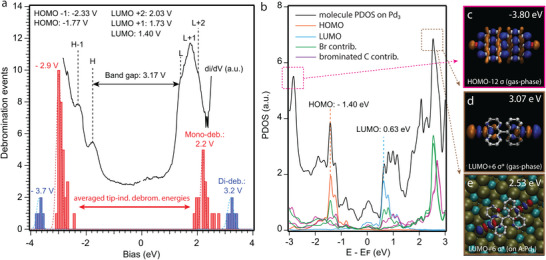
Average energies of tip‐induced debromination and DBBA's electronic structure. a) Red/blue bars: bias‐dependent histogram of debromination events for mono‐/di‐debromination reactions, respectively. Black tunneling spectra: representative tunneling conductance (DOS) of enantiomer R on the A:Pd_3_ surface. b) Simulated PDOS of DBBA molecule in gas phase marking HOMO/LUMO orbitals (in orange/blue) and contribution of bromine (green) and brominated carbon atoms (pink). c,d) Simulated spatial distribution of sigma anti‐bonding c) occupied and d) unoccupied DBBA orbitals in gas‐phase, and e) on A:Pd_3_ surface.

To further elucidate the details of the tip‐induced regioselective reactions, we study the relation of the voltage thresholds for the debromination with the position of the frontier molecular orbitals of DBBA as summarized in **Figure** **4**. The histogram of these thresholds for positive and negative voltage are shown in Figure 4a. The same initial tip‐sample position over the molecule was employed in all cases to avoid any influence of the tip position on the voltage threshold measurements (see Experimental Section for experimental details). The average tip voltage required to induce mono‐ and di‐debromination reflects a bias‐dependent asymmetry with respect to Fermi level. In more detail, both mono‐ and di‐debromination thresholds are lower at positive (+2.20 and +3.20 V) than at negative bias polarity (−2.90 and −3.70 V). Regarding this disparity, we detect a similar energy asymmetry in the frontier molecular orbitals of DBBA by means of scanning tunneling spectroscopy. Figure 4a includes a representative spectrum of DBBA's electronic structure on A:Pd_3_ surface featuring the first occupied and unoccupied molecular orbitals at −1.77 and +1.40 V, respectively. Given this correlation between activation energies and molecular bands, we simulate the density of states (DOS) of DBBA and its spatial distribution both in gas‐phase and on the A:Pd_3_ surface by DFT methods. Figure 4b shows the projected density of states (PDOS) of DBBA molecule adsorbed on the A:Pd_3_ surface. The resulting HOMO‐LUMO asymmetry with respect to Fermi level is in good agreement with the experimentally observed HOMO and LUMO. Beyond these first orbitals, the molecular DOS increases significantly at two molecular bands located close to −2.8 and +2.6 V, respectively. We relate this increase in the DOS to molecular orbitals of sigma bonding (σ) and anti‐bonding (σ*) character, respectively. Figure 4c,d shows the first occupied and unoccupied gas‐phase DFT σ‐orbitals. The preceding occupied and unoccupied orbitals found closer to the Fermi energy display in contrast π‐character (Figure S7, Supporting Information). Figure 4e shows the first unoccupied σ* orbital of DBBA adsorpted on A:Pd_3_, confirming the preservation of these orbitals upon adsorption. As further explained in the discussion section, we attribute the tip induced debromination to the injection (abstraction) of electrons into the anti‐bonding σ*‐orbital (from bonding σ‐orbital), whereby the C─Br is weakened and ultimately cleaved.

By thermal activation of the system at RT, complete mono‐debromination is experimentally observed. There are no discernable differences whether this process was performed by annealing cold‐deposited molecules or by RT deposition directly. This mono‐debromination of DBBA molecules at RT is analogous to previous reports on Ag(111)^[^ ^39^ ^]^ and Cu(110).^[^ ^40^ ^]^ Contrary to the regioselective tip‐induced debromination, we find two distinguishable stereo‐species of mono‐debrominated adsorbates in their threefolded orientations with equal distribution (Figure S8, Supporting Information). These two stereo‐species result from the debromination at one of the two inequivalent C‐Br sites. Therefore, we do not find any direct indication for regioselectivity in thermally induced debromination. Nevertheless, thermal mono‐debromination is further corroborated by the similar shape of these species with respect to their tip‐induced counterparts (Figure 3) and by the abundant presence of single bromine atoms dispersed along the A:Pd_3_ surface.

## 3 Discussion

To elucidate the tip‐induced mono‐debromination, we study the dependence of the excitation on the tunneling current and voltage (see Figure S9, Supporting Information). The former barely influences the reaction yield (≈ 10^−10^ reaction/electron), indicating a one‐electron process as commonly reported for tip‐induced dehalogenation on surfaces.^[^ ^42^, ^43^, ^44^ ^]^ By contrast, the bias dependence is highly non‐linear. Applying a sample bias voltage pulse of +2.25 V triggers the reaction with high yield, whereas no dehalogenation events are observed at +1.5 V. Such an energy threshold excludes participation of π‐character LUMO, positioned at +1.40 V. Besides energetic reasons, the bonding character of each orbital is crucial in order to determine its participation into the tip‐induced debromination. As observed for other halogenated aromatic adsorbates, bond cleavage seems to preferably occur by injection or abstraction of tunneling electrons into σ* anti‐bonding or σ bonding orbitals of the C─Br bond, respectively.^[^ ^45^ ^]^ As expected for aromatic molecules, these σ orbitals are spatially more localized along the DBBA structure than the spatially extended π orbitals. Importantly, the electron density of σ orbitals is located along the carbon and bromine atoms forming the C─Br bonds (white arrows in Figure S7, Supporting Information). In this way, once beyond the energy threshold of each orbital, electrons injected (abstracted) from the tip would populate (empty) these localized states at the C─Br bonds until inducing bond dissociation through C‐Br electron repulsion (depletion). The strong anti‐nodal shape of DBBA σ orbitals further support their anti‐bonding character. Electrons with lower energy than the threshold cannot participate in this process. By contrast, electrons with higher energy can still participate via the decay through higher energy π orbitals. The decay through these π orbitals is favored not only energetically, given the relative small energy gap between them, but also spatially given their broad extension over the molecule. The capacity of electrons to tunnel through these spatially and energetically delocalized π orbitals supports that the same C‐Br position is always activated in the tip‐induced debromination independently of the tip position over the molecule.

The robust regioselectivity of the tip‐induced debromination of conformer R on A:Pd_3_ evidences a substantial energy barrier difference between the two brominated positions stemming from a non‐equivalent interaction of these C─Br bonds with the local atomic arrangement of the substrate. We quantify the effect of this local surface environment by calculating the energy landscape of the debromination reaction for both halogenated positions by means of a nudged elastic band method. Figure S10, Supporting Information shows the energy of C─Br bonds as a function of the distance between both atoms. Our simulations depict an energetically favored scenario for the regioselective position, featuring an energy barrier (1.35 eV) significantly lower than its non‐regioselective counterpart (1.85 eV). This difference stemming from the different atomic environment under the to‐be‐broken C─Br bond: In the favorable case, the bond is over the gap between the two Pd trimers, in the other case on the Pd trimer itself hindering the detachment of the Br. Moreover, this lower energy barrier is related with a shorter C‐Br distance (3.05 Å) compared to the non‐favored C─Br bond (3.26 Å). This favorable energy landscape for the regioselective position justifies the (tip‐induced) bond selectivity of DBBA molecules on the A:Pd_3_ surface. We expect the origin of this robust bond selectivity to be related with the Ga atom in the second surface layer, which sits just below the regioselective C─Br bond (Figure 2e, bottom). Even though top‐layer Pd trimers are spatially more accessible for DBBA molecules at the surface, the dangling bond resulting from the debromination shows a clear preference to interact with second‐layer gallium atom. This preference can be rationalized from a chemical perspective, where Ga is known to be reactive in an oxidative (electron deficient) state, what would support the preference of the unpaired electron at the radical carbon position to engage with the electron‐lacking Ga atoms. By contrast, palladium is well‐known to be a relative noble element in general terms, commonly employed in jewelry manufacture and as catalyst in numerous chemical reactions. This dominant reactivity of second‐layer Ga atoms on chemical reactions on PdGa surfaces is further supported in literature by previous experiments reporting different reactivity for equivalent halogenated carbon atoms within the same adsorbate,^[^ ^46^ ^]^ and by etching procedures on PdGa crystals being mostly reactive with Ga atoms.^[^ ^47^ ^]^ In summary, the ability of Ga atoms to engage into chemical reactions with the organic adsorbates in this system would support not only the favorable energy landscape for the regioselective C─Br bond lying on top of these Ga atoms, but also the subsequent chemisorption of mono‐debrominated DBBA molecules upon this bond selective dehalogenation.

Contrary to the very pronounced regioselectivity of the R enantiomer, S lacks any selectivity and undergoes tip‐induced dehalogenation indiscriminately at one of its two halogenated positions (see Figure S11, Supporting Information). As depicted in Figure 2, the adsorption geometry of each enantiomeric form differs which is why R and S could be considered as diastereoisomers when the molecule‐surface configuration is considered. Although enantiomer S also places a C─Br bond over two adjacent Pd trimers (see Figure 2a, bottom), this bond does not sit directly over the second‐layer Ga atom, but over the bridge between a second‐layer Ga atom and a third‐layer Pd Atom. Therefore, we do not find the same favorable local configuration for the S on A:Pd_3_. As a result, the asymmetric substrate‐adsorbate interaction does not lead to drastic energetic asymmetries as observed for conformer R in the tip‐induced regioselective mono‐debromination.

The pronounced difference in debromination selectivity between the electronic tip‐induced and the thermally activated case deserves some further examination. At RT, the thermal mono‐debromination proceeds with a low reaction rate, needing hours to undergo full (mono‐)debromination of all DBBA molecules. This relative low reaction kinetics allows investigation of the system, with a mixture of intact and mono‐debrominated molecules. Figure S8, Supporting Information shows the system after 1 h at RT, where 70% of DBBA molecules are (thermally) mono‐debrominated. As the formation of the enantiopure phase precedes this partial dehalogenation, all intact DBBA molecules are R enantiomers. However, we observe two different stereo‐species of mono‐debrominated adsorbates (see Figure S8c,d, Supporting Information), strongly suggesting that the thermal debromination is not regioselective. Interestingly, tip‐manipulation can convert one species into the other by rotation and/or flipping with the C radical site resulting from the debromination not changing position (Figure S12, Supporting Information). Accordingly, both species are apparently anchored to the same adsorption site of the surface in these tip‐induced changes, supporting the expected organometallic bonding with the A:Pd_3_ surface upon bromine cleavage. Although these tip‐induced molecular motions are rarely observed while STM scanning at 5 K, by contrast at RT, these flipping events might occur at a high rate by thermal activation. In this way, even if the thermal C‐Br cleavage was to proceed regioselectively, this selectivity would quickly diffuse by the flipping events of both mono‐debrominated species, interchanging their stereo‐conformation with respect to the surface in these processes. Alternatively, we should consider the thermal mono‐debromination of DBBA molecules simply not being regioselective. The loss of regioselectivity can be explained by stronger vibrations and enhanced mobility of the molecules expected at higher temperatures. Accordingly, the well‐defined adsorption registry of DBBA molecules with A:Pd_3_ at 5 K, leading to the pronounced regioselectivity is lost. In conclusion, given the equal distribution of two different stereo‐species after thermal mono‐debromination and their capacity to turn one into the other through random flipping processes on the surface, we cannot conclude if the thermal mono‐debromination proceeds regioselectively as observed in the tip‐induced homologous reaction.

Besides these scarce rotation or flipping events of the mono‐debrominated species, a vast population of intact DBBA molecules also experiences flipping over the A:Pd_3_ surface as modeled in Figure 2 to achieve the near complete S to R conversion. First of all, this requires an energy difference between R and S enantiomers as with both conformers being energetically equivalent like on achiral substrates, this would result into an homogenous population of the surface.^[^ ^10^ ^]^ Second, the thermally activated adsorbate must be able to “flip over” the surface to change its conformation. A similar flipping process has been observed previously for planar (pro‐chiral) adsorbates^[^ ^25^ ^]^ on PdGa{111} surfaces. Here, the non‐planar DBBA molecule overcomes these structural restrictions with its flexible “scissor‐like” conformation, formed by two anthracene units linked by a single C─C bond acting as a rotation axis. This enables the variation of the dihedral angle between the subunits within a range limited by the steric hindrance of peripheral hydrogen atoms located in‐between the anthracenes units (Figure S4, Supporting Information). This innate hindered rotation characterizing DBBA's structural versatility represents a type of axial chirality known as atropisomerism.^[^ ^33^ ^]^ In fact, the influence of this structural flexibility of DBBA manifests significantly at different relevant aspects of on‐surface chemistry like adsorption, self‐assembly (Figure S1, Supporting Information), mobility or reactivity.^[^ ^28^, ^32^, ^39^, ^41^ ^]^ On achiral surfaces, the axial chirality of DBBA is known not only to preserve a chiral structural arrangement when self‐assembled, but to transfer this molecular chirality along a multistep synthetic process involving chiral polymers and finally chiral graphene nanoribbons.^[^ ^34^ ^]^ By contrast, on the chiral A:Pd_3_ surface, despite DBBA's versatility to adopt different structural conformations over the surface (Figure 2), the well‐defined adsorption with respect to the surface overrules any intermolecular interaction which would allow adsorbates' self‐assembly.

As previously discussed, all PdGa surfaces exert a combined effect between surface chirality and the so‐called ensemble effect.^[^ ^48^ ^]^ Although PdGa bulk chirality is preserved at all surface terminations, the particular atomic structure of each surface can drastically influence how this chirality interacts with molecular adsorbates.^[^ ^46^ ^]^ To further understand the chirality and ensemble effect in the enantioselective flipping process on the A:Pd_3_ surface, we deposit DBBA molecules on a different PdGa surface, namely PdGa:A(111)Pd_1_ (A:Pd_1_ surface for simplicity). A:Pd_1_ and A:Pd_3_ are opposed facets of the PdGa (A) crystal in the (111) direction. Hence, both surfaces share structural similarities such as the surface symmetry, surface lattice constant and electronic properties, but interestingly the Pd_1_ surface features equally‐separated Pd monomers, instead of trimers, as topmost layer. The substitution of trimers by single monomers renders a less dense top‐layer termination on the A:Pd_1_. Analogously to the A:Pd_3_ surface, cold‐deposition of DBBA on A:Pd_1_ surface at 90 K renders a weakly enantioselective adsorption scenario, yet with an excess of the S enantiomeric form (*ee* = 9%, see Figure S13, Supporting Information). Mild annealing to well below RT (at ≈160 K) also leads to a near enantiopure, but in this case S coverage (*ee* = 100%).

Despite the innate chirality of the A:Pd_1_ surface similarly dictates an asymmetric adsorption, the energetically favored enantiomer after thermal annealing turns interestingly to be S, contrary to A:Pd_3_ where we find R exclusively. Besides that each surface promotes an “inverse” DBBA flipping process, from enantiomer R to S or vice versa, the temperature‐evolution of the enantiomeric excess on each surface reveals interesting details. After cold‐deposition at 90 K on both surfaces, the enantiomeric excess is significantly higher on A:Pd_3_ (36%) than on A:Pd_1_ (9%). Upon mild annealing at 160–180 K, however, the enantiomeric excess on the A:Pd_1_ exceeds with near 100% the one observed for A:Pd_3_ with 63%. This suggests that the absorption energetics and the R ↔ S conversion dynamics are different for the two surfaces. Form our observations, the conversion process seems to proceed with a higher rate on the A:Pd_1_ surface. This might be expected from the lower density of the Pd_1_ top‐layer termination, compared to that of A:Pd_3_. Thus, anthracene subunits would find more space between monomers (than between trimers) to adapt their molecular structure, reducing the structural strain exerted by DBBA molecules along the series of surface‐assisted conformational changes involved in the flipping process. All considered, whereas the enantioselective adsorption behavior shows the dominant influence of the two chiral surfaces, the ensemble effect of each surface reflects differently on the structure of the DBBA molecules.

## 4 Conclusions

In conclusion, on both PdGa:A($\bar{1}\bar{1}\bar{1}$)Pd_3_ and PdGa:A(111)Pd_1_ surfaces we observe a chirality transfer from the substrate to DBBA molecules upon low‐temperature adsorption at 90 K, with an enantiomeric excess of 36% and 9%, respectively. Mild annealing well below room temperature results into the formation of enantiopure phases of individual DBBA molecules on both surfaces. The axial chirality of DBBA molecules engages with the chiral surface lattice upon adsorption, and this geometrical interplay relates closely with the intramolecular structural flexibility of the adsorbate. This enables DBBA molecules to change their enantiomeric form from R to S and vice‐versa through a surface‐assisted flipping mechanism of the two anthracene units around the central C─C bond. Thereby, adjusting their chiral conformation in a stepwise process to the chiral substrates. Importantly, we illustrate how the ensemble effect, that is, the local atomic structure and catalytic activity of this intermetallic compound, imposes a dominant bond selectivity in the tip‐induced dehalogenation. This process exemplifies the drastic influence that chiral PdGa crystals can apply on intramolecular activation energies changing the reactivity of the two C─Br bonds of DBBA which are chemically equivalent in the gas phase. As a result, the ensemble effect of catalytic PdGa crystal surfaces together with their chirality can finely tune the reactivity of adsorbates resulting into robust regioselective processes. Our study further underlines the potential of catalytic PdGa crystals not only for the formation of high‐quality chiral systems and 2D nanostructures at the macroscale with highest yields of enantioselectivity, but also to finely tune and study particular chemical effects of chiral‐dependent processes emerging from these systems for the fundamental understanding of structure‐properties relationships.

## 5 Experimental Section

### Experimental Methods

DBBA molecules (see reference^[^ ^28^ ^]^ for synthetic details) were sublimated from a home‐made evaporator directly onto PdGa and Au(111) surfaces, previously prepared by repeated cycles of sputtering (Ar^+^, 1 keV) and thermal‐resistive annealing at 925 K for PdGa, and 715 K for Au(111). Cold‐deposited samples were pre‐cooled down to 90 K prior deposition by a liquid nitrogen cooling pipe system installed along the sample manipulator.

STM images were acquired with a commercial low temperature STM from Omicron GmbH operated at 4.5 K in constant‐current mode (unless mentioned differently), with a base pressure below 1.0  ×  10^−10^ mbar.

In order to avoid any influence of the tip position on the quantitative determination of threshold voltages in the tip‐induced debromination, the STM tip was precisely positioned on top of the center of the molecule (between the two lobes) employing in each case the same scanning parameters (set‐point: ±2.0 V, 50 pA). Thereafter, the voltage was ramped up (0.05 V s^−1^) from ±50 mV in open‐feedback conditions until a clearly visible, single step‐like instability in the tunneling current is observed at certain voltage biases, indicating the debromination of the molecule. For evaluating the reaction yield of the tip‐induced mono‐debromination at positive bias, similar current instabilities were detected as a function of time, after positioning the tip precisely on top of the molecule and using the corresponding fixed voltage and current intensity values as open‐feedback conditions. Given the easily recognizable difference in STM shape between intact and debrominated species in this system (Figure S6, Supporting Information), tip‐induced reactions were unequivocally corroborated after the excitation in subsequent scanning images of the same surface area.

For the STM manipulation of enantiomer S into R illustrated in Figure S5, Supporting Information, the STM tip was positioned next to the molecule (≈2 nm) applying the manipulation parameters (V = 10 mV, It = 200 pA). The tip was moved afterward over the molecule in a linear trajectory (≈5 nm) keeping the same manipulation parameters in open‐feedback conditions until the detection of the flipping process in consecutive images.

### Theoretical Methods

All simulations were performed using the CP2K^[^ ^49^ ^]^ atomistic simulations software package on the AiiDALab^[^ ^50^ ^]^ platform. The geometry optimizations of Figure 1d,e, Figure 3g–j, Figure 4b–e were performed within the framework of density‐functional theory (DFT)^[^ ^51^ ^]^ using the PBE^[^ ^52^ ^]^ parametrization of the generalized gradient approximation (GGA). The substrate‐molecule systems were modeled in the framework of the repeated slab scheme,^[^ ^53^ ^]^ such that a simulation cell contains 18 atomic layers of PdGa along the [−1‐1‐1] direction, the adsorbed DBBA molecule, and 30 Å of vacuum. Furthermore, van der Waals (vdW) dispersion interactions are present in all simulations according to Grimme's D3 method.^[^ ^54^ ^]^ The STM image simulations of Figure S2, Supporting Information, were obtained from the wavefunction output files as a result of these structure relaxations. The input and output files of the simulations are contained in the supplementary information of this work.

The minimum‐path‐energy model simulating the flipping of enantiomer R into S (Figure 2) was performed using the nudged elastic band (NEB)^[^ ^55^ ^]^ method to first determine the path between two S and S' (Figure 2a,b), and then again from S' to R' over the transition state (Figure 2b–d), and finally from R' to conformer R (Figure 2d,e). To perform the NEB calculations, the initial and final states of each respective chain were optimized using the DFT‐PBE‐GGA method. The transition state was then located using a NEB calculation with an interpolated path between the optimized S' and R' conformations. This approach allowed for the identification of the minimum‐energy path between the two stable conformations and the transition state. The energy difference of 0.26 eV between conformer R' (Figure 2d) and enantiomer R (Figure 2e) is the difference between the converged value of the total energy after the structure relaxation.

The dependence of DBBA's energy with respect to the dihedral angle between anthracene units in gas phase (Figure S4, Supporting Information) was obtained using the constrained replica chain method,^[^ ^56^ ^]^ whereby the collective variable is defined as follows. First, a line is drawn through the midpoints of the benzene rings on each anthracene unit: two non‐coplanar lines result. These two lines form an angle which is used as the collective variable.

The simulated energy barriers for the debromination of both halogenated positions as function of the C─Br bonding distance (Figure S10, Supporting Information) were computed in each case by using 16 geometries previously generated using a constrained replica chain, and then further optimized using a NEB, with the constraint being the Br‐C distance. In this way, even though successive configurations between the initial equilibrium configuration peak at their respective transition state, the Br species does not “fall” back to rejoin with the now‐radical C. The final resting point of the debrominated Br was chosen, in alignment with the experimental observations, to be atop the center of the closest lying top‐layered Pd trimer (see Figure 3g,h).

### Statistical Analysis

For the evaluation of all enantiomeric excess (*ee*  = |*R* − *S*| *100/(*R* + *S*)), and the thermal mono‐debromination yield of DBBA adsorbates at RT as a function of time (Figure S8, Supporting Information), individual molecules were counted in different STM images. The data collected on these observations were adjusted to a Poisson distribution. Any adsorbate of ambiguous shape, or which adsorption registry could be altered by the presence of nearby undesired adsorbates or surface defects, was not considered for the analysis. A sample size of 1824 individual adsorbates (within 54 STM images) on A:Pd3, and 527 individual adsorbates (within 37 STM images) on A:Pd1, were considered for the evaluation of the enantiomeric excesses on each surface. A sample size of 162 individual adsorbates (within 5 STM images) were considered for the evaluation of the thermal mono‐debromination yield.

To evaluate the energy location of the molecular bands of DBBA on A:Pd3 and Au(111) with respect to Fermi level, a sample size of 50 and 23 spectroscopic files were considered, respectively. To evaluate the average energy required to tip‐induce mono‐/di‐debromination on A:Pd3, a sample size of 57 voltage ramping files were considered. These statistical results were adjusted to a Gaussian distribution, and average results were expressed as mean ± 1 standard deviation. Data outliers were visually identified in histogram charts.

For the evaluation of the tip‐induced mono‐debromination dependency on voltage and current intensity at positive bias (Figure S9, Supporting Information), a sample size of 314 tunneling current files were considered. Linear regression analysis were applied to the electron yield to conclude the one‐electron behavior of the tip‐induced debromination.

Igor Pro 8 software (Wavemetrics), including home‐made built‐in scripts, was employed for the visualization and analysis of all STM images and spectroscopic files.

## Conflict of Interest

The authors declare no conflict of interest.

## Supporting information

Supporting Information

## Data Availability

The data that support the findings of this study are openly available in https://archive.materialscloud.org/ at https://doi.org/10.24435/materialscloud:3f‐1k, reference number 2023.
