# Supplementary material for: Asymmetric Molecular Adsorption and Regioselective Bond Cleavage on Chiral PdGa Crystals
Source: Adv Sci (Weinh). 2024 Feb 14;11(16):2309081. doi: 10.1002/advs.202309081 (PMC11040335; doi:10.1002/advs.202309081)
Supplement: Supplementary file 1 — Supporting Information [file ADVS-11-2309081-s001.pdf]

ADVANCED SCIENCE
Open Access

Supporting Information

for *Adv. Sci.*, DOI 10.1002/advs.202309081

Asymmetric Molecular Adsorption and Regioselective Bond Cleavage on Chiral PdGa Crystals

Nestor Merino-Diez, Raymond Amador, Samuel T. Stolz, Daniele Passerone, Roland Widmer and Oliver Gröning*

Asymmetric molecular adsorption and regioselective bond cleavage on chiral PdGa crystals

Nestor Merino-Diez, Raymond Amador, Samuel T. Stolz, Daniele Passerone, Roland Widmer, Oliver Gröning*

Nanotech@surfaces Laboratory, Empa – Swiss Federal Laboratories for Materials Science and Technology, Überlandstrasse 129, 8600 Dübendorf (Switzerland)

Corresponding author: roland.widmer@empa.ch

SUPPORTING INFORMATION:

S1. RT-deposition of DBBA molecules on Au(111): self-assembled islands and electronic structure

We deposit DBBA at RT on Au(111), as an achiral surface reference. Once that step edges are saturated, DBBA molecules self-assemble into islands along the fcc regions, either as linear rows along the herringbone ditches (Supp. Figure 1c) or as irregular islands at domain boundaries (Supp. Figure 1b). Among these latter irregular aggregates, some DBBA molecules self-assemble in circular aggregates of pentamers or hexamers (blue arrows .in panel *b*), like previously reported on Ag(111) for high molecular coverages[1]. A minor portion of molecules pins to surface 'elbow' dislocations, presumably fixing the nucleation point of the self-assembled islands. In contrast to the well-defined adsorption of DBBA molecules on the A:Pd_3 surface, the assembly of adsorbates on Au(111) is dominated by intermolecular interactions overruling the relatively weak adsorbate-substrate interaction in this case. DBBA molecules are known to form armchair graphene nanoribbons on Au(111) upon thermal annealing[2]. Contrary to A:Pd_3 samples at RT where all molecules are mono-debrominated, DBBA molecules are intact on Au(111) after RT-deposition. According to the d-band model introduced by Nörskov and coworkers[3], this stronger catalytic effect of Pd_3 surface for the mono-debromination is expected because of similar reactivity of PdGa with respect to Ag(111)/Cu(111)[3], where DBBA molecules are known to undergo partial/complete debromination at RT[4]. Additionally supporting this stronger adsorbate-substrate interaction on A:Pd_3 with respect to Au(111), we observe a smaller band gap on the former (3.17 V, Figure 4a) than on the latter (4.02 V).

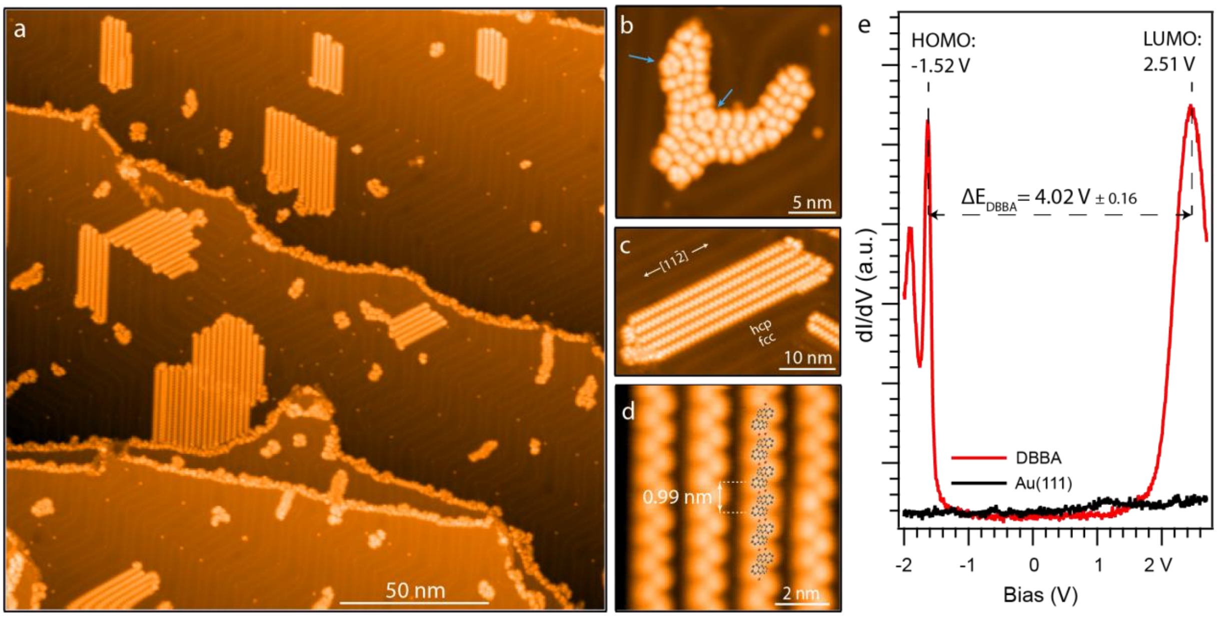

Supp. Figure 1: DBBA molecules after RT-deposition on Au(111). **(a)** Representative STM image of the system with low molecular coverage. **(b-d)** Examples of self-assembled islands (b) irregularly arranged at domain boundaries, (c,d) and linearly arranged in rows along the fcc ditches of the Au(111) surface reconstruction. Blue arrow indicates a pentamer/hexamer. Models of self-assembled molecules are superimposed in *d* as visual guide. **(e)** Representative tunneling spectrum of DBBA on Au(111) [Tunn. parameters (V,I_t): a) -1.50 V, 20 pA; b) -1.00 V, 50 pA; c) -1.00 V, 12 pA; d) -1.50V, 12 pA; e) set-point: 1.00 V , 20 pA.].

S2. Simulated STM images of intact and debrominated DBBA molecules on the A:Pd_3 surface:

We perform simulated STM images of the intact and debrominated DBBA molecules on the A:Pd_3 surface by DFT, easing their visual identification in experimental STM images. The detailed chiral motifs of the simulated species fit precisely with those observed experimentally, and remark the apparent (counter-) clockwise helicity of the anthracene subunits of intact DBBA molecules imaged by STM.

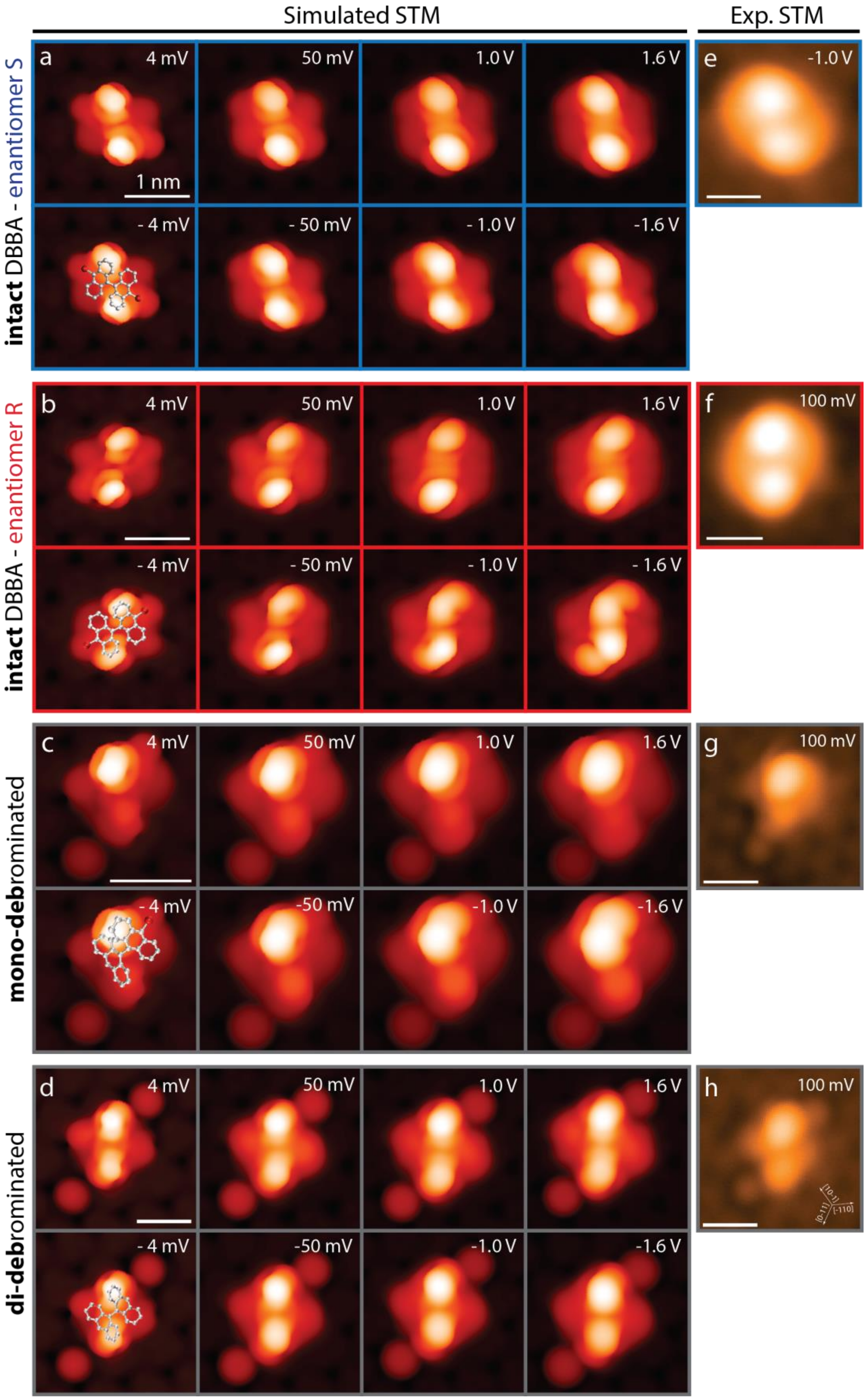

Supp. Figure 2: DFT-simulated STM images of intact and debrominated DBBA species. **(a-d)** Simulated STM images on A:Pd_3 surface at (top) positive and (bottom) negative bias polarity of **(a)** intact enantiomer S and **(b)** enantiomer R, **(c)** mono- and **(d)** di-debrominated DBBA molecules. Molecular models superimposed as visual guide. **(e-h)** Corresponding experimental STM images of each specie. All white scale bars correspond to 1 nm. [Tunn. parameters: e) V= 1.0 V, I_t = 30 pA ; f,g,h) V = 100 mV, I_t = 30 pA].

S3. Additional images of enantiopure phase of enantiomer R on A:Pd_3.

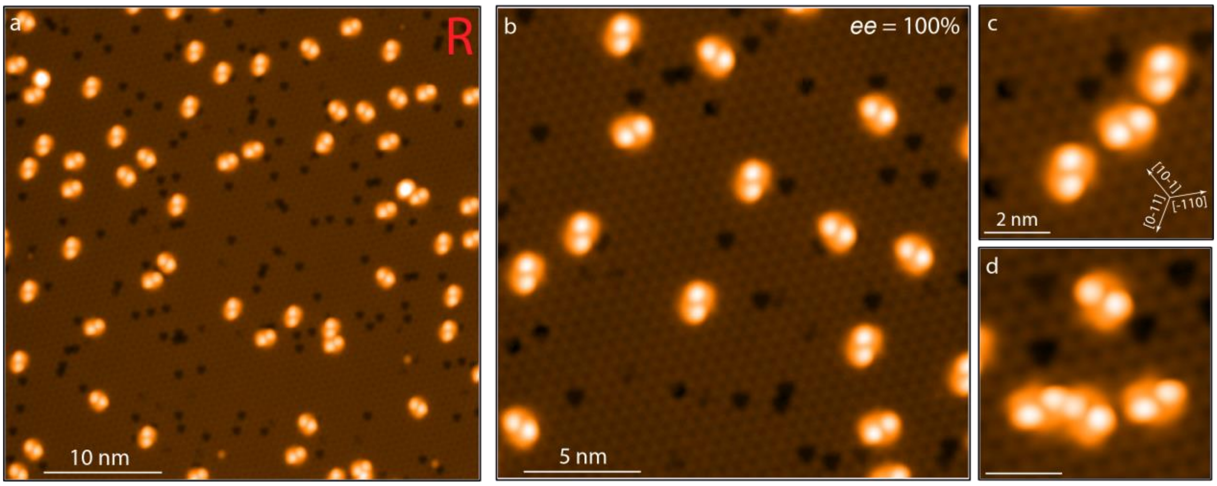

Supp. Figure 3: more representations of enantiopure phase of enantiomer R on A:Pd_3 surface. [Tunn. parameters: V = 50 mV, 20 pA].

S4. DBBA in gas phase: forbidden anti-parallel rotation of anthracene units

Simulations using constrained geometry optimizations illustrate the DBBA internal energy as a function of the dihedral angle between anthracenes. The parabolic growth of DBBA energy when anthracene units are brought to the same plane supports molecular fragmentation happening before any successful anti-parallel rotation of anthracene units, as reported previously for similar molecules[5]. The difference in energy between the angle 0° and 180° is due to the initial conditions of the optimization with the central dihedral fixed at a certain value. The energy profile is however largely symmetric around the minimum. Constrained optimizations were performed with the software ORCA[6] using the hybrid functional B3LYP and the split valence polarization Karlsruhe basis set (def2-SVP) (A triple-zeta basis set was also tested but the differences were found to be negligible)[7].

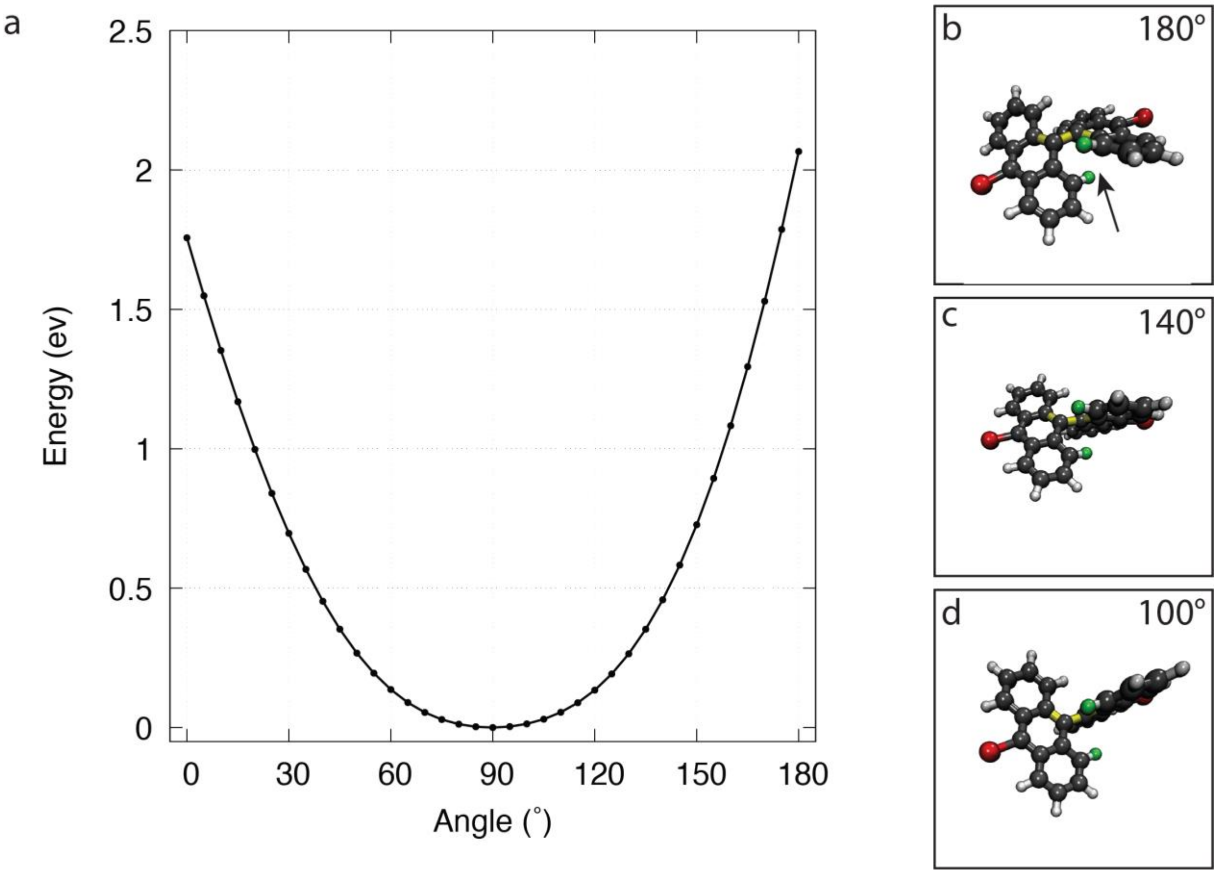

Supp. Figure 4: DBBA in gas phase. **(a)** Energy as a function of dihedral angle between anthracene units. **(b-d)** DBBA models for dihedral angles of (b) 180°, (c) 140° and (d) 100°. The hydrogens causing the most steric repulsion for high torsional angles are highlighted in green, and the bonds defining the torsional angle in yellow. Black arrow in panel *b* indicates a high steric hindrance situation between (overlapping) H atoms located in-between anthracenes.

S5. Tip-induced on-surface enantiomer flipping on A:Pd_3

On-surface flipping of single molecules can be attained by tip-manipulation methods, enabling the in situ interconversion of enantiomers. This tip-induced single-molecule process supports the collective flipping of all enantiomer S into R by thermal means (see Figure 1).

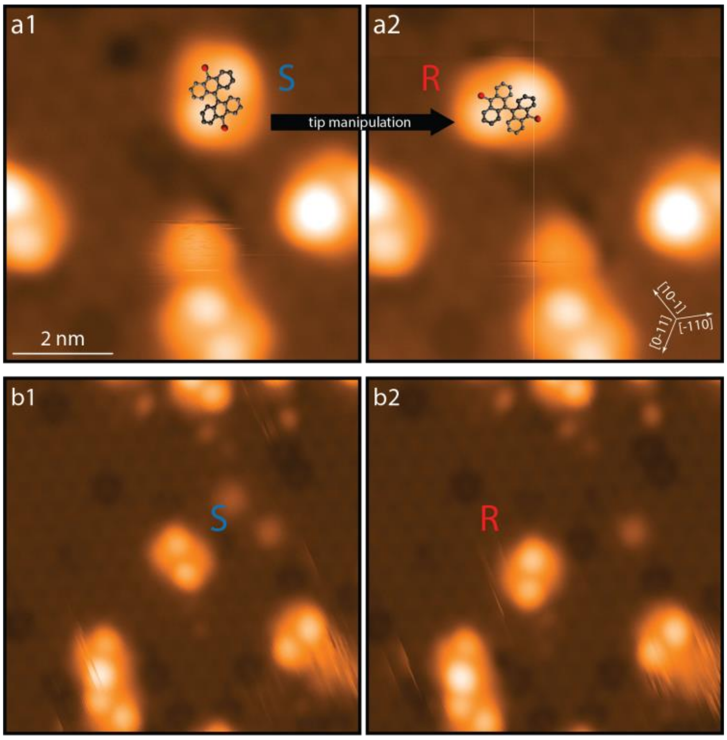

Supp. Figure 5: Tip-induced on-surface enantiomer flipping on A:Pd3. **(a,b)** STM images illustrating examples where (left) enantiomer S is transformed into (right) enantiomer R inducing a rotation of the molecule. [Tunn. parameters: V=1.00 V, I_t=20 pA ; Tip-manip. parameters: V=10 mV, I_t=200 pA].

S6. Examples of tip-induced mono- and di-debrominated molecules

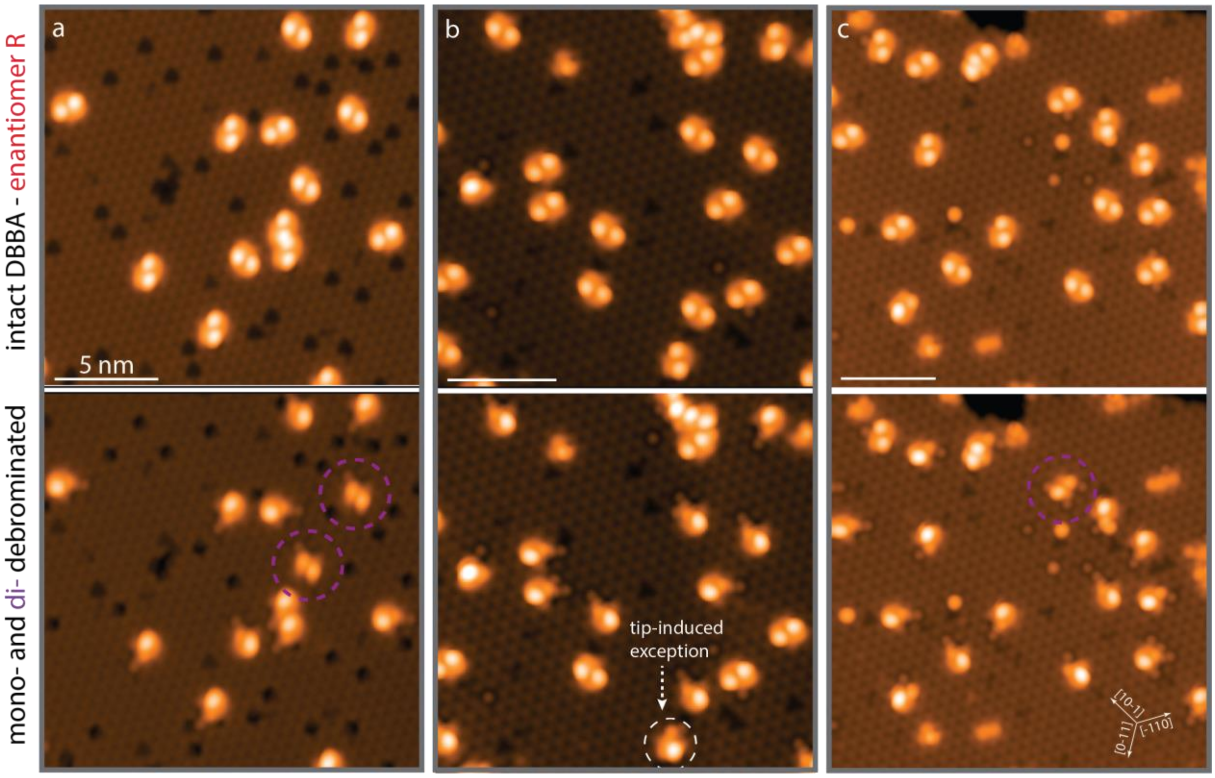

Supp. Figure 6: Additional examples of tip-induced mono-and di-debrominated DBBA molecules (white circles). Purple circles indicate di-debrominated DBBA molecules. White circle indicates one of the scarce examples observed of tip-induced mono-debromination at the non-favored C-Br bond for conformer R. [Tunn. parameters (V, I_t): a) 50 mV, 12 pA; b,c) 100 mV, 50 pA].

S7. Molecular orbitals of DBBA molecule in gas phase.

We simulate the molecular orbitals of DBBA molecules in gas-phase by DFT methods in order to evaluate the *sigma* and *pi* character of each orbital. As expected for an aromatic molecule, a strong *pi* orbital shape dominates the electronic structure of DBBA molecule in general terms, locating the electron density in parallel planes along (above/below) each anthracene subunit. Beyond the first molecular orbitals, which are completely dominated by *pi*-like DOS, a clear *sigma*-like DOS defines the (un)occupied orbitals LUMO+6 and HOMO-12, respectively. The nodal planes characterizing these sigma orbitals depict clearly their anti-bonding character[8].

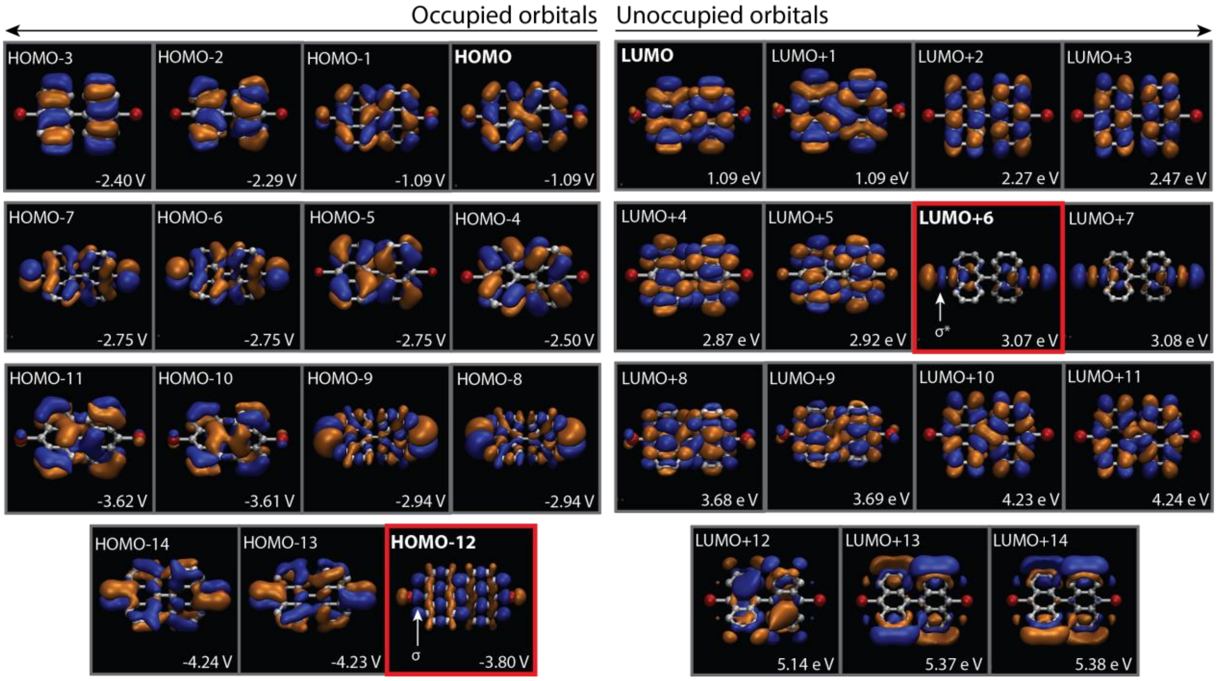

Supp. Figure 7: DFT-simulated molecular orbitals of DBBA in gas phase. **(left)** Occupied and **(right)** unoccupied orbitals. Red squares highlights the lowest energy (un)occupied orbitals featuring DOS with sigma anti-bonding character. White arrows indicate DOS located in nodal planes between carbon and bromine atoms.

S8. Thermal mono-debromination of DBBA on A:Pd_3 surface at RT.

Either annealing cold-deposited samples, or depositing DBBA molecules directly at RT, results into a homogenous distribution of two different mono-debrominated in its three-fold rotations. One of these mono-debrominated adsorbates (column *c*) matches in shape and orientation to their tip-induced counterparts (Figure 3).

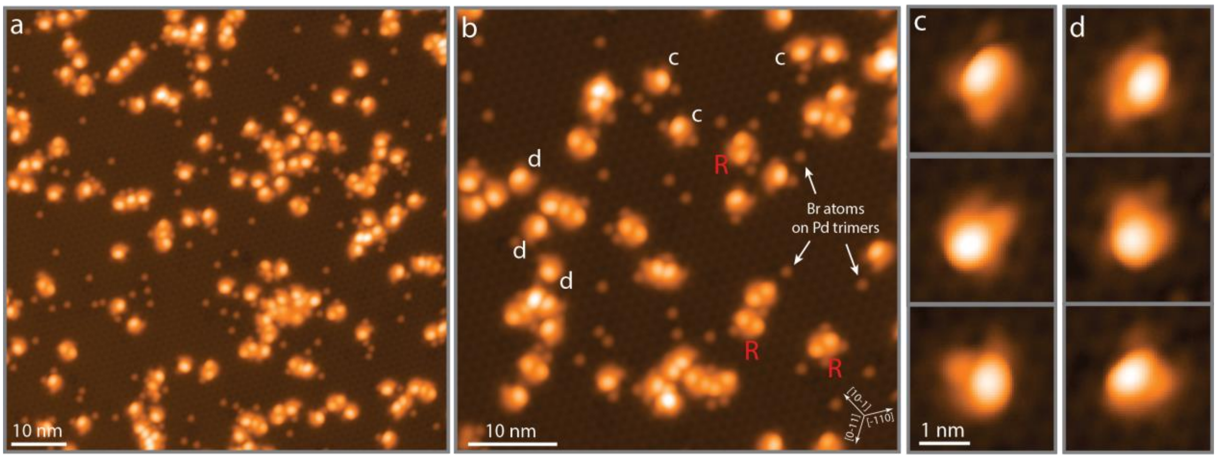

Supp. Figure 8: Thermal mono-debromination of DBBA on A:Pd_3 surface at RT. **(a,b)** Representative STM images of the system after 1 hour at RT, and **(c,d)** mono-debrominated DBBA molecules found in homogeneous population. Mono-debrominated adsorbate in column *c* resembles in shape and orientation to their tip-induced counterparts [Tunn. parameters (V, I_t): a,b) 1.0 V, 40 pA; c-h) 50 mV, 50 pA].

S9. Dependence of tip-induced mono-debromination on current and voltage (positive bias)

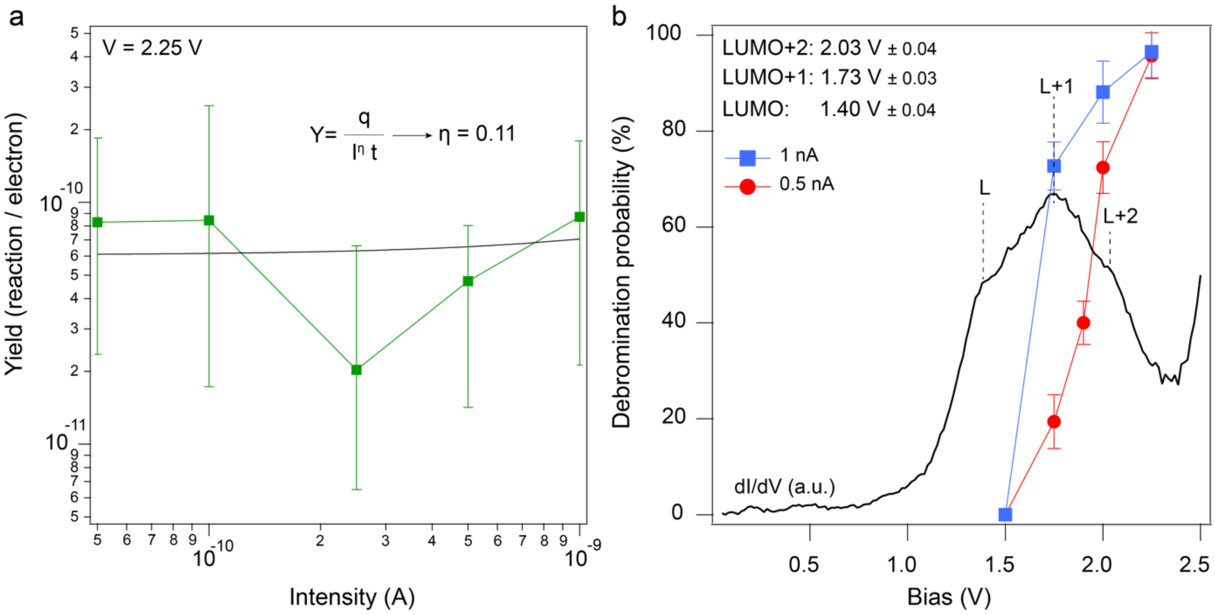

Supp. Figure 9: Tip-induced debromination dependence on current intensity and voltage. **(a)** Reaction yield as a function of the current intensity for a bias voltage of 2.25 V. **(b)** Red/blue: bias-dependent probability of successful debromination. Black: representative tunneling spectrum of first unoccupied DBBA orbitals on A:Pd_3 surface.

S10. Debromination energy landscapes for both C-Br bonds of enantiomer R.

Starting from identical initial intact relaxed configurations and Br-C distances (1.93 Å) for both the left and right debrominations, we employ the nudged elastic band method to generate 16 geometries to obtain an energy vs. Br-C distance profile. The final configuration for each was chosen as that in which the detached Br atom rests atop the Pd trimer as observed experimentally, see Figs. 3g, 3h. The Br-C distance at the transition state (TS) was simulated to be 3.05Å (left debromination) and 3.26Å (right debromination).

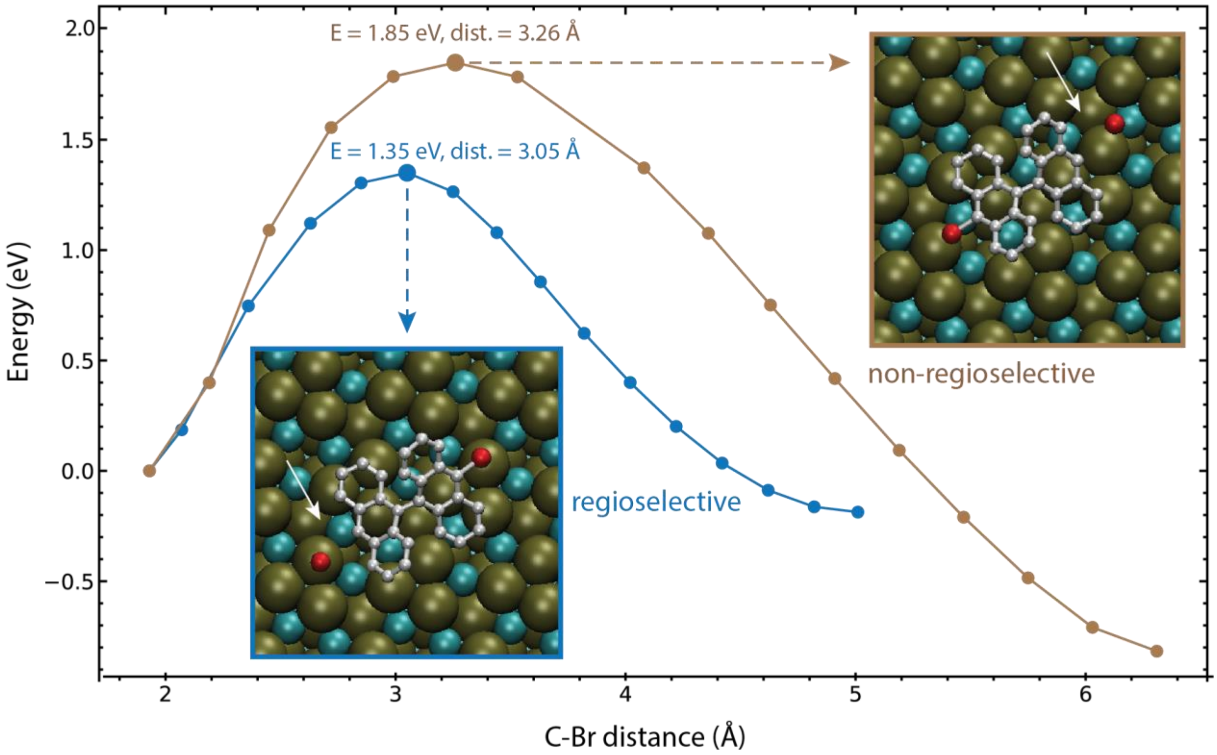

Supp. Figure 10: Energy barriers for both halogenated positions as a function of C-Br distance for conformer R. (Blue/brown) for the regio- and non-regioselective C-Br bonds. Insets: models representing the transition state configurations for both paths, emphasizing the different environment (a Pd-Pd gap for the lower barrier, part of the Pd trimer for the higher barrier) causing the difference in activation energy.

S11. Non-regioselective tip-induced debromination of enantiomer S on A:Pd_3

Tip-induced debromination is tested for the enantiomer S on A:Pd_3 but, contrary to enantiomer R, no regioselectivity is observed this time. Images below illustrate two different enantiomer S with the same orientation (for an easier comparison) where debromination is tip-induced at different brominated positions.

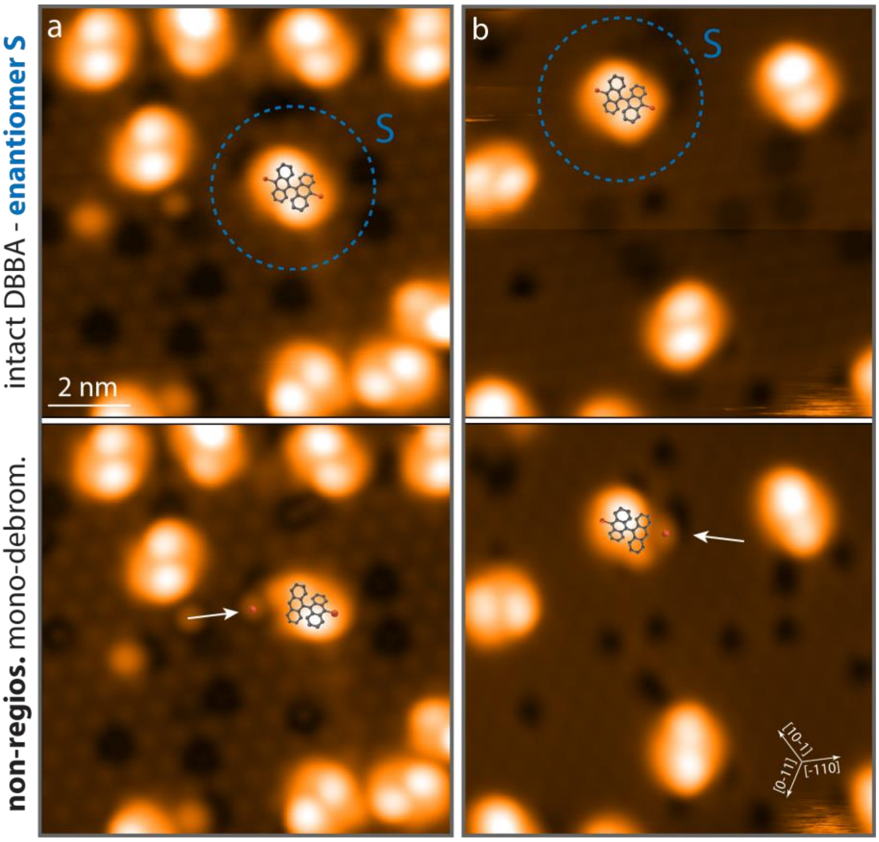

Supp. Figure 11: Non-regioselective tip-induced debromination of enantiomer S on A:Pd_3. **(a,b)** Examples of enantiomer S (with the same orientation, for an easier comparison), **(top)** before and **(bottom)** after tip-induced debromination. White arrows indicate debrominated positions. [Tunn. parameters (V,I_t): a) -1.00 V, 100 pA; b) 1.00 V, 20 pA]

S12. Tip-induced rotation and flipping of mono-debrominated molecules on A:Pd_3.

We eventually observe tip-induced changes in mono-debrominated DBBA molecules while scanned by STM. These dehalogenated adsorbates rotate, or even flip, seemingly anchored to the surface, supporting a chemisorption scenario.

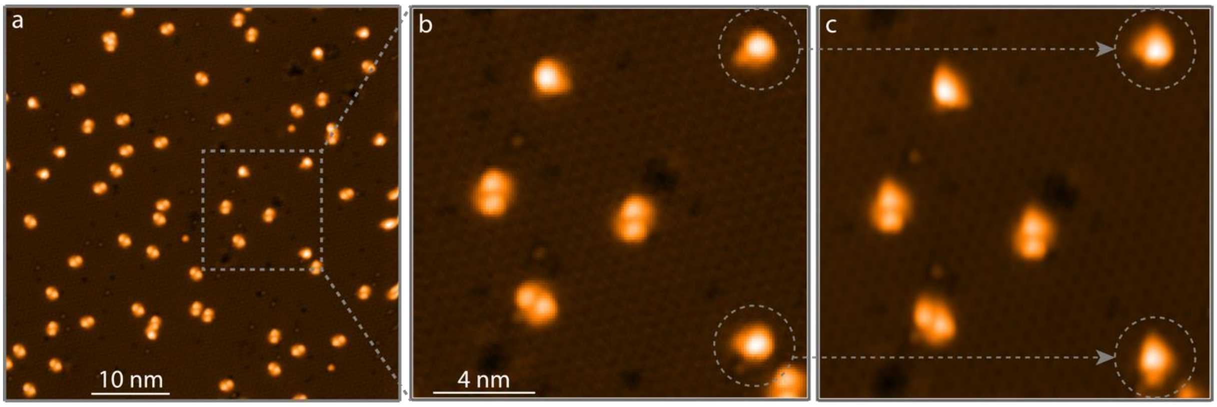

Supp. Figure 12: Tip-induced motion of mono-debrominated DBBA molecules chemisorpted on A:Pd_3**. (a)** Representative STM images of the system after partial thermal debromination. **(b,c)** Examples of rotated mono-debrominated DBBA molecules after consecutive scanning. [Tunn parameters (V,I_t): a-d) 1.20 V, 20 pA.].

S13. Temperature-dependent enantioselective adsorption of enantiomer S on A:Pd_1 surface.

DBBA molecules are cold-deposited at 90K on A:Pd_1 surface for comparison. The A:Pd_1 surface commonly presents numerous last-layer vacancies, this is, missing Pd_1 monomers at the top[9]. As a result, some DBBA molecules are adsorbed within these vacancies (white arrows). These vacancy-adsorbed adsorbates show a different STM appearance, where one anthracene unit adopts a more perpendicular tilting with respect to the surface as suggested by their prominent single lobe. This characteristic appearance is easily distinguishable from two-lobed DBBA molecules adsorbed on a non-defective area. Therefore, vacancy-adsorbed DBBA molecules were excluded from any further analysis. As portrayed in the figure below, the enantioselectivity in the adsorption process evolves as well into an enantiopure phase (*ee* = 100%) in favor of enantiomer S on A:Pd_1 when the system reaches 160 K, thus similarly featuring a temperature-dependent behavior as enantiomer R on A:Pd_3.

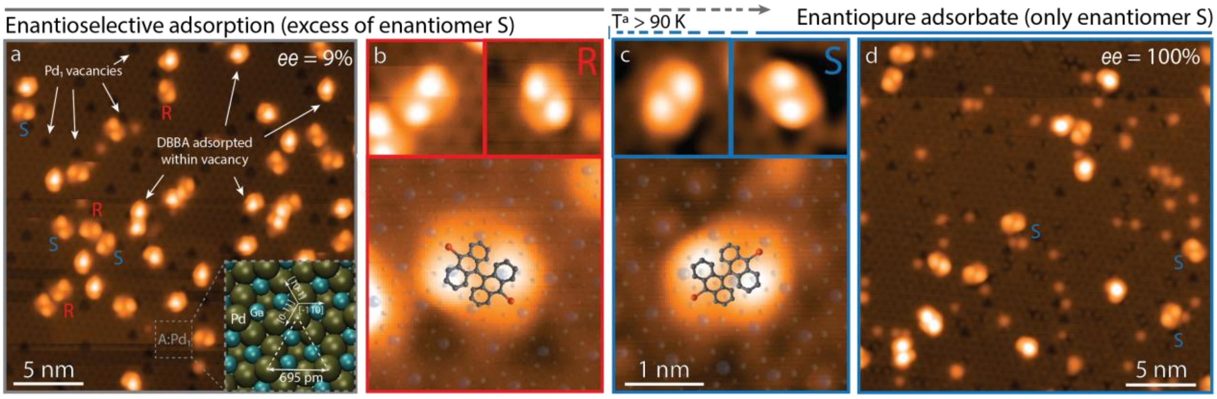

Supp. Figure 13: Temperature-dependent enantioselective adsorption of enantiomer S on A:Pd_1 surface. **(a)** Representative STM image after cold-deposition of DBBA at 95K. White arrows indicate top-layer Pd_1 vacancies and DBBA molecules adsorbed within these vacancies, respectively. **(b)** Enantiomer R and **(c)** enantiomer S in its three favored orientations on A:Pd_1 surface **(d)** STM image of enantiopure phase formed at a temperature above 90 K. [Tunn. parameters: a-d) V =1.50 V; I_t = 50 pA].

References

1. Shen, Y. *et al.* Chiral Self-Assembly of Nonplanar 10,10′-Dibromo-9,9′-bianthryl Molecules on Ag(111). *Langmuir* **33**, 2993–2999 (2017).
2. Cai, J. *et al.* Atomically precise bottom-up fabrication of graphene nanoribbons. *Nature* **466**, 470–473 (2010).
3. Nørskov, J. K., Bligaard, T., Rossmeisl, J. & Christensen, C. H. Towards the computational design of solid catalysts. *Nature Chemistry* **1**, 37–46 (2009).
4. Simonov, K. A. *et al.* Effect of Substrate Chemistry on the Bottom-Up Fabrication of Graphene Nanoribbons: Combined Core-Level Spectroscopy and STM Study. *J. Phys. Chem. C* **118**, 12532–12540 (2014).
5. Merino-Díez, N. *et al.* Transferring axial molecular chirality through a sequence of on-surface reactions. *Chem. Sci.* **11**, 5441–5446 (2020).
6. Neese, F. The ORCA program system. *WIREs Computational Molecular Science* **2**, 73–78 (2012).
7. Hellweg, A. & Rappoport, D. Development of new auxiliary basis functions of the Karlsruhe segmented contracted basis sets including diffuse basis functions (def2-SVPD, def2-TZVPPD, and def2-QVPPD) for RI-MP2 and RI-CC calculations. *Phys. Chem. Chem. Phys.* **17**, 1010–1017 (2015).
8. Timm, M. J. *et al.* Contrasting Efficiency of Electron-Induced Reaction at Cu(110) in Aliphatic and Aromatic Bromides. *J. Am. Chem. Soc.* **142**, 9453–9459 (2020).
9. Prinz, J. Surface Science Investigations on Structure and Binding Centers of Intermetallic PdGa Surfaces. (EPFL, 2014).
